# A Mediterranean Diet Model in Australia: Strategies for Translating the Traditional Mediterranean Diet into a Multicultural Setting

**DOI:** 10.3390/nu10040465

**Published:** 2018-04-09

**Authors:** Elena S. George, Teagan Kucianski, Hannah L. Mayr, George Moschonis, Audrey C. Tierney, Catherine Itsiopoulos

**Affiliations:** 1 Department of Rehabilitation, Nutrition and Sport, La Trobe University, Health Sciences 3, Kingsbury Drive, Bundoora, VIC 3086, Australia; T.Kucianski@latrobe.edu.au (T.K.); H.Mayr@latrobe.edu.au (H.L.M.); G.Moschonis@latrobe.edu.au (G.M.); A.Tierney@latrobe.edu.au (A.C.T.); C.Itsiopoulos@latrobe.edu.au (C.I.); 2 School of Exercise and Nutrition Sciences, Deakin University, Building J, 221 Burwood Hwy, Burwood, VIC 3125, Australia; 3 School of Allied Health, University of Limerick, Castletroy, Limerick V94 T9PX, Ireland

**Keywords:** Mediterranean diet, dietary intervention, diet, nutrition, translation, non-alcoholic fatty liver disease, cardiovascular disease

## Abstract

Substantial evidence supports the effect of the Mediterranean Diet (MD) for managing chronic diseases, although trials have been primarily conducted in Mediterranean populations. The efficacy and feasibility of the Mediterranean dietary pattern for the management of chronic diseases has not been extensively evaluated in non-Mediterranean settings. This paper aims to describe the development of a MD model that complies with principles of the traditional MD applied in a multiethnic context. Optimal macronutrient and food-based composition was defined, and a two-week menu was devised incorporating traditional ingredients with evidence based on improvements in chronic disease management. Strategies were developed for the implementation of the diet model in a multiethnic population. Consistent with the principles of a traditional MD, the MD model was plant-based and high in dietary fat, predominantly monounsaturated fatty acids from extra virgin olive oil. Fruits, vegetables and wholegrains were a mainstay, and moderate amounts of nuts and seeds, fish, dairy and red wine were recommended. The diet encompassed key features of the MD including cuisine, biodiversity and sustainability. The MD model preserved traditional dietary components likely to elicit health benefits for individuals with chronic diseases, even with the adaptation to an Australian multiethnic population.

## 1. Introduction

There is a substantial body of evidence to support the efficacy of the Mediterranean Diet (MD) in chronic disease prevention and management [1]. In this regard, results from several intervention studies support the efficacy of a MD for individuals with cardiovascular disease (CVD) as well as a broad range of associated disease states, including metabolic syndrome, type 2 diabetes mellitus (T2DM), cognitive function decline, depression and anxiety, autoimmune diseases, cancer and non-alcoholic fatty liver disease (NAFLD) [2,3,4]. As inflammation drives insulin resistance and oxidative stress [5,6], the anti-inflammatory and antioxidant properties of the MD pattern are proposed to slow the progression of some of the oxidative damage associated with the aforementioned clinical conditions [7,8].

Some of the proposed benefits of the MD pertain to the dietary pattern as a whole. This encompasses overall macro and micronutrient composition, the variety of foods included as well as the combination, preparation and consumption of these foods which contribute to the synergistic effect of the food matrix [9]. The combination of foods and how these are traditionally prepared is often referred to as cuisine, and this is a key element of the MD that is likely to contribute to added health benefits [10].

The majority of clinical trials which have assessed the efficacy of the MD have been conducted in Mediterranean populations, where this dietary pattern and these types of foods are familiar [1]. Embedding the principles of a MD in non-Mediterranean populations worldwide with different habitual food cultures remains a great challenge. Australia is a culturally diverse nation, with almost half the population (49%) having either been born overseas or with at least one parent born overseas [11]. Given the high prevalence of chronic disease in Australia, and the scientific evidence base of the MD, assessing the efficacy of the diet for the prevention and management of these diseases in this population group is warranted. However, given the heterogeneity around the definitions and interpretations of what constitutes a MD intervention, researchers need to be transparent about the exact methods and dietary prescriptions used to ensure reproducibility and translation of favourable effects for other research studies and to drive practice change [12].

The current paper provides the rationale and process undertaken for the development of a MD model intervention in two clinical trials, currently underway. Protocols for each study are presented elsewhere Papamiltiadous et al. [13] and Itsiopoulos et al. [14]. In this context, the specific aims of the present study were (1) to identify the nutrient composition profiles of previous MD interventions delivered to Mediterranean and Australian populations; (2) to identify the key dietary and food-based components within the MD (with evidence-based health benefits) and to use these to develop key food intake recommendations; (3) to develop a two-week meal plan based on the nutrient composition profile and the key food intake recommendations identified in the previous steps; and (4) to assess the barriers to translatability of this dietary pattern to multiethnic Australian populations with chronic disease and to develop strategies to overcome these.

## 2. Methodological Steps to Address Each Specific Aim

**Figure 1:**
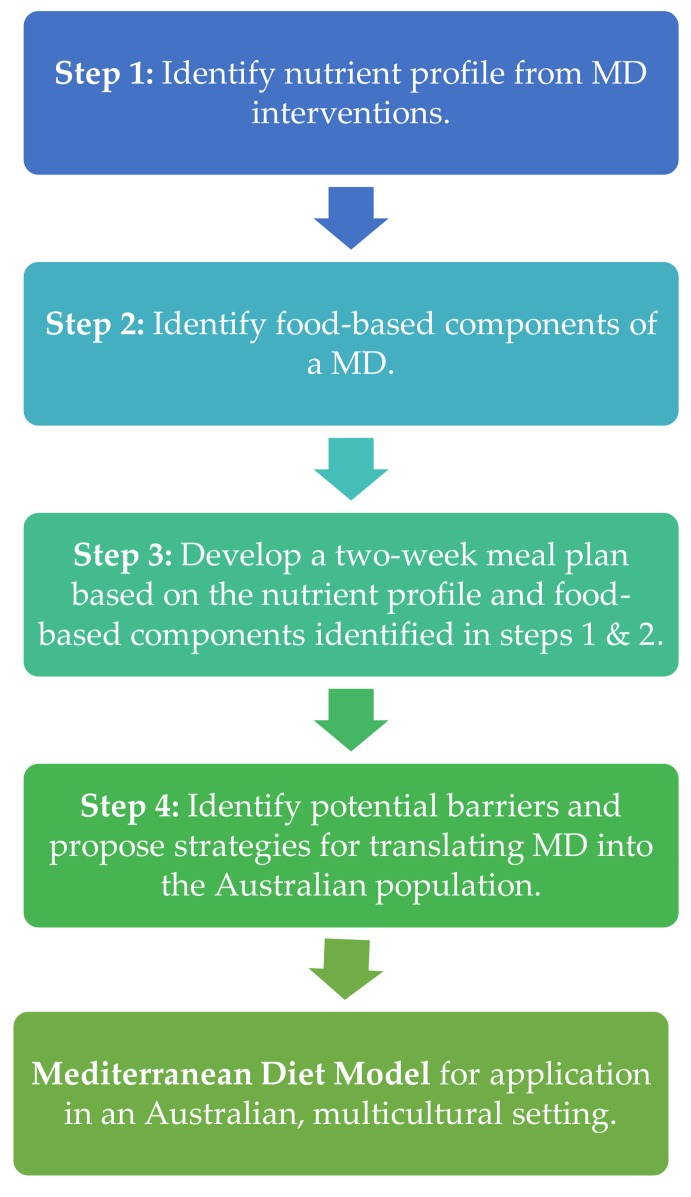
A schematic summarising the key methodological steps taken to develop a Mediterranean Diet model for an Australian multicultural setting. MD: Mediterranean Diet.

A MD intervention for provision in an Australian, multiethnic population with diagnosed NAFLD and CVD, undergoing clinical trial conditions, was developed using the below steps which are summarized in Figure 1.

### 2.1. Step One: Identification of the Nutrient Composition Profile of Previous MD Interventions Delivered to Mediterranean and Australian Populations

In determining the key nutrients required to achieve a MD, it was important to establish a clear definition of a ‘MD pattern’ using a strong evidence base. This included using MD interventions that have successfully translated this dietary pattern and achieved health benefits. There have been a number of trials published, which refer to and apply a MD intervention; however, the nutrient composition across the literature remains inconsistent [12]. Therefore, the authors used the Cretan MD as the definition for this MD model, which was determined using published data from trials based on the archetypal traditional Cretan (Greek) MD [15]. These dietary practices have been the most pivotal in demonstrating prevention and management of chronic disease [16,17,18].

A comprehensive assessment of MD studies, including seminal MD trials in Mediterranean regions and smaller clinical trials implemented in the Australian context, was conducted. Nutrient composition data is depicted in Table 1. From this assessment the nutrient compositions for this intervention were derived and are discussed below.

Table 1 captures the nutrient composition from trials where substantial benefits were demonstrated with the prescription of the MD. Benefits were defined as improvements in outcome measures in a range of chronic diseases or where feasibility in an Australian population was shown.

Of note, the landmark LYON diet heart study was clearly the most impressive secondary prevention trial using a Mediterranean style diet to prevent secondary myocardial infarcts; however, the dietary intervention model of this trial was not used to inform our intervention model due to major differences in fat type. The LYON heart study had a heavy reliance on high alpha-linolenic acid (ALA) canola oil and margarine which are not documented traditional fats used in the MD [22]. Clinical trials encompassing traditional MD principles that have been carried out in non-Mediterranean regions, namely Australia, were feeding trials that provided all meals to participants for the study duration [4,19]. These trials demonstrated ‘proof of concept’ that positive health outcomes can be achieved for chronic disease states in Australian populations with consumption of a MD. Ongoing provision of complete meals is not achievable and is cost prohibitive in clinical trials and does not adequately represent the feasibility of implementing this dietary pattern in a free living population. Therefore, the Australian dietary intervention developed and described herein encompasses the traditional MD patterns for application in free living participants [13].

### 2.2. Step Two: Identification of the Key Food-Based Components of a MD with Strong Published Evidence of Health Benefits

Using the nutrient profile derived in step one, food components with health promoting effects were used to formulate a meal plan. When translating optimal levels of essential nutrients into food-based recommendations, references in Table 1 were used as a guide, as were the Hellenic dietary guidelines [23]. It is worth noting that since the development of this MD model intervention model, the Hellenic dietary guidelines have been updated. Cross referencing with published MD food group recommendations, labelled ‘commandments’, was also carried out [24]. Determination of key food-based recommendations and a review of the evidence-based mechanisms surrounding these dietary components were conducted; these are summarised in Table 2.

For both steps one and two, there was also consideration of other evidence-based, disease specific dietary recommendations. For example, for CVD patients, the National Heart Foundation guidelines were incorporated, and for NAFLD patients, alcohol recommendations were kept to the lower end of the suggested range. This was important to ensure that the MD model dietary model did not was consistent with established clinical guidelines for these medical conditions.

### 2.3. Step Three: Development of a Two-Week Meal Plan Based on the Nutrient Composition Profile and the Key Food Intake Recommendations Identified in the Previous Steps

Based on steps one and two, a two-week meal plan, suitable for an Australian multiethnic population was developed (Figure S1). The meal plan was based on the macro and micronutrients and food-based components consistent with a traditional MD. These meal plans were analyzed using a standard food analysis program, Foodworks 7™, to ensure nutrient composition was consistent with the desired profile as identified in step one. The two-week meal plan also incorporated other key elements of the MD highlighted in step two, including combinations and preparation of foods described as part of the MD cuisine.

### 2.4. Step Four: Identification of Potential Barriers and Proposal of Strategies for Translating MD into the Australian Population

The studies presented are summarized in Table 1 and Table 2 and were used to determine the perceived feasibility of the intervention through highlighting potential barriers for the translation of a MD into the Australian population. To overcome these barriers, a theoretical framework encompassing a SWOT (strengths, weaknesses, opportunities, threats) analysis was conducted. This was carried out to identify barriers that were likely to occur and embed strategies within the MD model to overcome these. This SWOT analysis was conducted alongside the experience and expertise of practising dietitians and researchers to ensure appropriate strategies were embedded into the intervention with the aim of addressing the perceived barriers identified.

## 3. Practical Strategies Related to Each Specific Aim

### 3.1. Nutrient Composition Profiles of Other MD Interventions Delivered to Mediterranean and Australian Populations

The rationale for the MD intervention model, including the composition, specific ingredients and cooking methods, is discussed throughout this section. As described in step one of the previous section, a prospective cohort trial as well as four other clinical trials [4,19,20,21] were used to document the ideal nutrient components of the MD, and their reported nutrient data is summarised in Table 1. The single observational study and the four clinical trials identified in step one were used to derive desirable macro and micronutrient ranges to inform the dietary prescription of the MD model.

The application of this Mediterranean diet model will determine whether this diet can be translated to the Australian, multiethnic population and whether a MD pattern is sustainable in the long term. Furthermore, the MD model intervention is being tested within two clinical trials that are currently underway [13] to determine the efficacy, feasibility and sustainability of delivering this intervention in Australian cohorts with chronic diseases, such as Coronary Heart Disease (CHD) and NAFLD.

### 3.2. Macronutrient Profile of the MD Model

As well as the nutrient compositions described in Table 1, this section provides an overview of the macronutrient composition and corresponding foods for the MD model intervention. The MD model intervention is a ‘high fat’ diet, with fat comprising, at the macronutrient level, between 35% and 45% of energy intake. At least 50% of the energy from fat is from monounsaturated fatty acids (MUFAs) and the remaining energy contribution comes from polyunsaturated fatty acids (PUFAs) and saturated fatty acids (SFAs). Protein contributes 15–20% of total energy, and carbohydrates contribute 35–40%. This MD intervention also includes moderate amounts of alcohol which may contribute up to 5% of total energy, which is within the recommendations set by the Australian National Health and Medical Research Council (NHMRC) [25]. The importance of the specific nutrient profiles within the MD model and how they impact on chronic disease risk factors are described below.

#### 3.2.1. Fats

##### Monounsaturated Fatty Acids

This MD model intervention emulates the traditional MD in that it is high in MUFAs, mainly due to the daily consumption of Extra Virgin Olive Oil (EVOO), the predominant culinary fat used in the diet. While there are other oils that are classed as high MUFA, they do not contain the same level of MUFA as EVOO. Importantly, EVOO contains an abundance of polyphenols which are thought to drive the anti-inflammatory and antioxidant benefits attributed to the MD diet [26,27].

##### Polyunsaturated Fatty Acids

PUFAs are also a key nutrient encompassed in the MD intervention. These are sourced from a variety of foods, including nuts, seeds, olive oil and fish. Long chain omega-3 fatty acids, namely eicosapentaenoic acid (EPA) and docosahexaenoic acid (DHA), are particularly abundant in marine dietary sources. The plant-derived essential PUFA, alpha linoleic acid, is derived from staple dietary components, such as wild edible greens and nuts and seeds. These omega-3 rich PUFAs are critical for achieving a more favourable omega-6 (*n*-6) to omega-3 (*n*-3) ratio within the diet. A traditional Cretan (Greek) MD is characterised by a 2:1 ratio, while Western diets are closer to 20:1 [28]. An elevated *n*-6:*n*-3 ratio mediates detrimental vascular changes and limits anti-inflammatory processes, which likely exacerbates oxidative stress, increasing the risk and severity of chronic diseases [29]. The MD model diet developed for our dietary interventions and presented in this manuscript achieved a favourable *n*-6:*n*-3 ratio close to 3:1, reflected in Table 2.

##### Saturated Fatty Acids

SFAs constitute a small proportion of the overall MD model intervention—less than a 10% contribution to total energy consumption. The small SFA component of the diet is derived from staple MD components, such as EVOO, nuts and seeds and animal-based products, such as yogurt and small amounts of meat. SFA-rich products such as processed foods and large amounts, or frequent consumption of, animal products are not a feature of the traditional MD, nor were these foods incorporated into our MD model [30].

#### 3.2.2. Carbohydrates

Carbohydrates contribute between 35–40% of energy in the MD model intervention. The focus is on whole grains which are processed minimally and never refined. Traditionally, these included sourdough bread, potatoes, rice and pasta. The accessibility of ‘true’ sourdough bread is not readily available in Australian supermarkets. Thus, soy and linseed bread was recommended as an alternative which also served to increase dietary alpha-linoleic acid. The traditional MD was consumed *ad libitum*; therefore, portions are not a key feature of the MD model intervention. However, given the quantities of carbohydrate consumed in Australian populations [31], to achieve the desired macronutrient contribution, examples of appropriate portions for carbohydrate-based foods were provided within the principles of the MD intervention to moderate intake.

#### 3.2.3. Protein

The MD model is a predominantly plant-based diet, and animal proteins are consumed in small amounts [28]. Thus, a lot of dietary protein is sourced from a variety of plant-based protein sources, including legumes, lentils, nuts and seeds. The MD also includes moderate consumption of fish and animal protein, including dairy, eggs and white meat, and red meat is recommended for less frequent consumption. The traditional MD included infrequent and small portions of white meat and even less red meat. However, to ensure acceptability, the MD model intervention allows a maximum quantity of 450 g/week (white and red meat varieties). In the MD model intervention, recommended dairy sources are predominantly fermented, including yogurt and white cheese, especially feta (Table 2). Of note, low fat/skim or light dairy alternatives were not traditionally part of a MD; however, modern dairy production does not follow traditional methods, whereby some full fat options contain additional fat as cream to enhance ‘creaminess’. Therefore, low fat options were used in the MD intervention (analysis described in Table 3) to achieve the desired SFA composition as per Table 1 and to ensure that the Australian National Heart Foundation (NHF) Guidelines were maintained, as appropriate. This was especially important given that participant cohorts targeted for the application of the MD model intervention were at risk of, or had CVD [32].

#### 3.2.4. Alcohol

Alcohol, traditionally from red wine, is included and recommended to be consumed with meals in the MD model. However, individuals not previously consuming alcohol were not encouraged to commence drinking. The quantity recommended is one to two standard glasses of red wine per day, which is within the Australian NHMRC alcohol recommendations of <20 g ethanol per day [25]. The maximum contribution of energy from alcohol included in the composition is approximately 5% of total energy. In some instances, alcohol was not recommended. For example, patients who had progressed NAFLD were advised to avoid alcohol due to the increased risk of hepatocellular carcinoma [33].

### 3.3. Identification of the Key Food-Based Components of a MD with Strong Published Evidence of Health Benefits

The food-based recommendations developed in step two are presented in Table 2. Scientific literature was used to support the development of these recommendations, and identification of components which include key mechanisms that drive optimal health outcomes for the management of chronic diseases are also highlighted in Table 2.

#### 3.3.1. Food Groups

The food group recommendations in Table 2 are based upon documented MD ‘commandments’ [24]. Table 2 highlights these adapted food-based recommendations which were designed to be easy for participants to understand and interpret. Education material outlining these dietary recommendations and how they could be applied has been designed to be provided to participants in the initial dietary consultation, with accompanying pictures to aid translatability (Figures S2 and S3). Table 2 also highlights the proposed mechanisms and evidence-based benefits of each recommendation—these were not included in patient resources.

#### 3.3.2. Cuisine

The term cuisine refers to the method of cooking which is often characteristic to a country or region [10]. Cooking includes the fusion of different ingredients, which has been shown to have additional nutritional benefits compared with its isolated and/or uncooked counterparts. Specifically, the addition of olive oil to tomatoes during cooking considerably increases the absorption of lycopene (a carotenoid linked to reduced rates of certain cancers and heart disease) [10]. Furthermore, the antioxidant capacity of salads has been assessed with and without the addition of aromatic herbs. Lemon balm and marjoram (1.5% (*w*/*w*)) increased antioxidant capacity by 150% and 200%, respectively [66]. In terms of salad dressings, the combination of EVOO and wine or apple vinegar gave the greatest increase in antioxidant capacity [66].

Given the benefits of cuisine in the MD pattern, it was important that cooking and preparation methods were captured in this MD model diet intervention. When providing dietary recommendations concerning cuisine for participants in this intervention there were two key areas embedded into the dietary intervention design, and these were emphasised during consultations: (1) herbs and spices and (2) cooking. These are elaborated upon below.

#### 3.3.3. Herbs and Spices

Culinary herbs and spices are a part of the traditional MD, where they are used to flavour most dishes, and many are thought to have medicinal properties based on their ability to heal [28]. It is thought that the enhancement and depth of flavour resulting from the addition of herbs and spices contributes, at least in part, to the enjoyment and palatability of MD meals and may therefore encourage sustainability. The evidence in modern science for the benefits of the phytonutrients found in culinary herbs and spices continues to grow. Polyphenols have been associated with improved health outcomes due to the antioxidant capacity of these phytonutrients and thus, an increased quantity and improved bioavailability of polyphenols through dietary cuisine is desirable [27]. It is well established that herbs and spices are a concentrated source of polyphenols; for example, the polyphenol contents in 100 g of both oregano and carrot are 935 vs. 58 mg gallic acid equivalents (GAE), respectively [67,68]. It is also well accepted through other dietary guidelines, such as those endorsed by the Australian NHF, that flavour enhancement using herbs and spices can reduce the addition of excessive salt (sodium) to meals, which is associated with hypertension [32]. The element of cuisine was a key consideration within the development of the MD intervention to ensure replication of these health benefits. To encourage the addition of herbs and spices in meal preparation, recipes and meal plans were provided to illustrate how to cook with these ingredients.

#### 3.3.4. Cooking

In order to achieve the benefits of combining ingredients and replicating the benefits of a ‘cuisine’ from the MD dietary pattern, traditional cooking practices are encouraged. In addition, cooking skills and cooking itself are linked to improved health outcomes [69,70]. Encouraging participants to cook facilitates dietary changes becoming part of lifestyle and behaviour, assisting in sustaining the changes, and thus achieving benefits.

**Figure 2:**
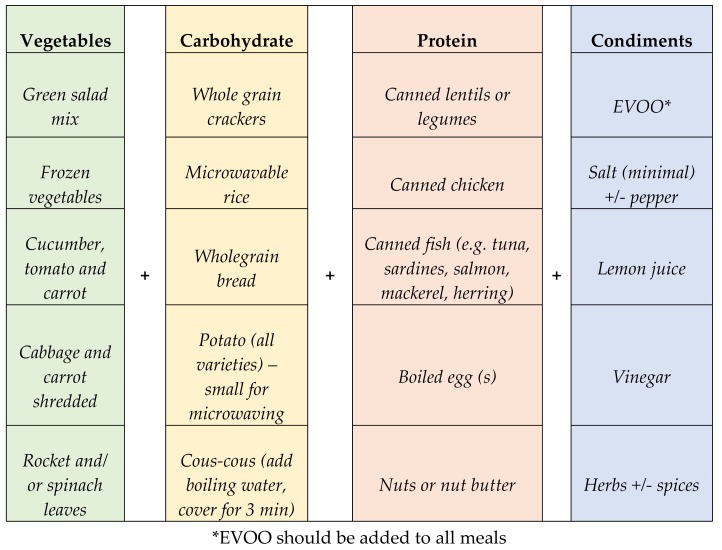
A summary of the MD inspired ‘Cook free’ meals based on the resource provided to study participants. EVOO: extra virgin olive oil.

Of note, many participants who are involved in clinical trials are either not willing to, or unable to prepare foods and cook meals for a range of reasons. The need for fast and convenient foods is well recognised in today’s busy lifestyles. To accommodate for such lifestyles, resources were provided to assist participants to ‘assemble’ meals with choices that required minimal preparation. These convenient options enabled nutritious choices, while retaining key elements of the MD model without the need for expansive cooking skills or equipment. These quick and easy meal options were designed within a resource that could be provided to all participants but was emphasised to those who had limited cooking skills or for meals such as lunch at work where there was limited time and/or access to cooking equipment. Figure 2 highlights how these types of nutritious, MD-inspired cook-free meals were modelled in the resource developed for provision to participants (Figure S4)). This resource was supplied to participants as part of a toolkit to enable them to implement the principles of this diet within their own diet and lifestyle.

#### 3.3.5. Eating Together

A key aspect of the traditional MD pattern is the emphasis of eating together. Food pyramids which depict MD often include a component showing people sharing meals [30]. This element is featured because it is the essence of the MD culture. It is thought that the table acts as a unifier and gives a sense of community [30,71,72]. This ideology was captured within our dietary intervention by recommending social interaction such as family meals or eating with others.

#### 3.3.6. Being Mindful

A key part of the traditional MD is the notion of eating to appetite. Thus, to assist in facilitating the inclusion of this *ad libitum* approach, elements of mindfulness are included as a part of the MD intervention delivery. As well as eating with others, participants are encouraged to practice mindfulness between and during meals, avoid electronic devices and screen time during meals, and be more aware of what they are eating through slower eating, chewing thoroughly and understanding their hunger and fullness cues [73].

#### 3.3.7. Biodiversity and Seasonality, Local and Eco Friendly

Including a predominantly plant-based varied diet is a key component of the MD pattern. In addition, the MD traditionally focused on local, accessible and seasonal produce. This is important, as accessible produce is (1) environmentally and eco-friendly due to reduced emissions in production, storage and transportation; (2) often provides a more nutrient-dense food source; and (3) is usually more abundant and therefore affordable [30,74]. With regard to the practical application of this aspect of the dietary intervention, food variety and choices are adapted for the Australian context. The example provided in Figure 2 extrapolates the key points addressed during a dietary consultation to achieve practical application of biodiversity and seasonality in the Australian context.

**Figure 3:**
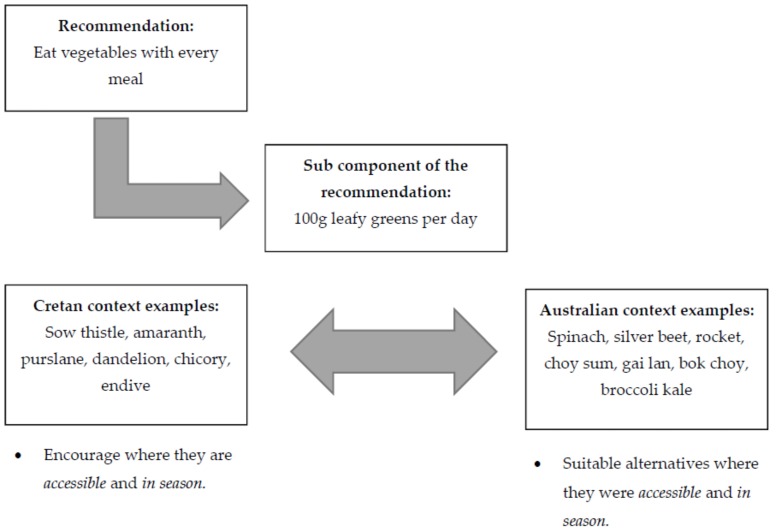
An example which models the practical dietary application of a recommendation from the traditional Cretan MD in an Australian context. Biodiversity and seasonality are considered.

The example shown in Figure 3 for traditional MD and modifications for the Australian context is by no means exhaustive. It simply showcases one example of flexible implementation of the dietary intervention. This flexibility allows for seasonal and accessible produce in the Australian context and accommodates different cultural preferences including what is familiar and acceptable to individuals. This substitution example is important to ensure that the diet model is feasible and translatable into multiethnic populations.

### 3.4. A Two-Week Meal Plan Based on the Nutrient Composition Profile and the Key Food Intake Recommendations Identified in the Previous Steps

The nutrient ranges described in Table 1 in conjunction with the key principles of a MD described in Table 2 guided the development of the MD model intervention and the two week meal plans discussed in this section. The two-week meal plan was analysed in Foodworks™ version 7 and the macro- and micronutrients that were derived from these menus are reported in Table 3. These meal plans are available in Figure S1. There were three main meals and three snacks provided per day. A recipe book [24] detailing the preparation of all the meals and a shopping list (Figure S5) accompanied the meal plans. So, while the meal plan was designed to model optimal consumption of MD, composition was assumed to deviate from this based on individuals’ preferences, satiety, cooking skills, culture, etc. The recommended meal pattern is an important component of the dietary model and is described here explicitly to clarify the purpose of each recommendation.

#### 3.4.1. Breakfast

For the first meal of the day, the diet plan aimed to incorporate wholegrains and high contents of MUFAs and antioxidants while considering what was easily accessible. The options ranged from porridge with fruit as well as including more traditional components, such as honey and cinnamon. Soy and linseed bread with either chopped tomatoes, onion, herbs, EVOO and lemon juice or eggs with stewed tomatoes or avocado were also included. Greek style yogurt with fruit, honey, cinnamon and nuts were also incorporated into the meal plan. Herbal tea or coffee were included daily. Less traditional components, such as using low fat dairy, soy and linseed bread, were substituted to ensure an optimal fat ratio for the meal plan was reached.

#### 3.4.2. Lunch/Dinner

Lunch meals were intended to be high in vegetables, wholegrains and MUFAs. Options ranged from soy and linseed bread, fish and salad or roast vegetables and feta cheese with EVOO, lentil/legume-based soups, or leftover dinner meals. Smaller meals were also accompanied by a piece of fruit. Canned fish, for example, is a less traditional component of the meals which was included to meet desirable fatty acid profiles while maintaining the convenience often desired in Western societies.

Dinner meals were also high in vegetables, wholegrains and MUFAs. Most of the meals at dinner required some cooking, but, as previously mentioned, convenient meal resources could be used in place of this. Furthermore there were tips on how main meals could be altered or made more convenient. The main meals at dinner included fish, small portions of white meat twice per week, and red meat, which was limited to once per week. There were at least two vegetarian days per week and at least two lentil dishes per week. Meal plans were designed so that meals could be moved around between meal times, days and weeks to suit individual preferences and time commitments.

#### 3.4.3. Snacks

Snacks primarily composed of Greek yogurt, fresh or dried fruit and/or raw unsalted nuts providing a source of antioxidants as well as wholegrains, MUFAs and/or protein. There was an allowance for a small, homemade sweets after dinner twice per week. Snacks provided an opportunity to include important elements of the MD, such as fermented dairy and healthy fats from nuts, and were not just ‘fillers’.

### 3.5. Potential Barriers and Proposed Strategies for Translating MD into the Australian Population

When designing the MD intervention for Australians, a key consideration was whether the diet would be feasible in a non-Mediterranean population. It is generally easy to replicate desirable macronutrient contributions such as those described for the MD as macronutrients are broad and non-specific. However, the task becomes more multifaceted when foods and their combination through cuisine is considered, to replicate the synergistic effects of the food matrix. When drawing on more customary recipes from various cuisines, each meal has its own unique flavour profile based on the region from which it originates. It is therefore important to model how the key elements of cuisine within the MD could be replicated across a variety of culturally-specific dishes. Table 4 provides an example of a traditional Cretan (Greek) Mediterranean dish called Fasolatha, and models popular multicultural alternatives which include similar key ingredients and combinations in cooking. This example demonstrates that macronutrients and foods, even when combined as part of cuisine from the MD, can be captured and translated across other traditional recipes. This demonstrates that the MD principles shown to improve health, can be replicated for a multicultural setting, which is both important and relevant due to the multiethnic landscape of Australia. This also ensures flexibility can be achieved through individualised dietary advice that can accommodate individual eating preferences. This substitution therefore enables the MD to be translatable across multiple ethnic groups.

## 4. General Overview and Future Implications

This paper described the nutrient and food-based components of a traditional Mediterranean diet using data from seminal trials and a cohort trial including dietary intakes of people from Crete in the 1950s, considered the archetypal traditional Mediterranean diet. This information was used to model the development of a MD model for specific use in two multiethnic cohorts with chronic diseases: CVD [14] and NAFLD [13].

The MD model intervention includes specific dietary prescription, including a breakdown of macro and micronutrients and food components. This allows for transparency when reviewing literature and thus optimal analysis of dietary adherence and translation into other research trials and clinical practice. This belief around ensuring transparency was inherent in the modelling of this paper through the development and design of the MD model intervention and showed that the components of the Mediterranean cuisine [20,30] could be maintained across a number of different cultural cuisines. There is a strong rationale for assessing the efficacy of the MD in a Western, multicultural population where there is a need to identify a superior dietary pattern that can prevent and manage the growing epidemic of chronic diseases. This dietary pattern should also be assessed for its ability to be achieved and sustained by individuals. Furthermore, the MD model described herein has been developed for application within free living populations. This is an important strength of this dietary intervention model because theoretically, if adopted by individuals, it is more likely to be sustained. Sustainability is an outcome that is critical for the management of chronic diseases.

Having comprehensively reviewed the key nutrients, foods and mechanisms which facilitate health benefits, the development of this MD model intervention is anticipated to be comparable to interventions delivered in the Mediterranean basin. The MD model intervention is consistent with the traditional MD, being a predominantly plant-based diet high in dietary fat, predominantly from MUFA from EVOO consumption. The MD model intervention reported in this paper also features moderate amounts of fermented dairy, fish and white meat with minimal amounts of red meat. White and red meat portions are higher than those consumed as part of a traditional MD, where an allowance of 450 g/week was developed in accordance with successful clinical trials described in Table 1 and Table 2. This resulted in a slightly higher total protein recommendation in the MD model (Table 3) compared with percentages reported in the traditional MD counterpart. Furthermore, there is an increased amount of prescribed α-linoleic acid and total long chain *n*-3s included within the MD model intervention model in an effort to improve the n6:n3 ratio consumed, which is indicated in clinical practice guidelines for CVD.

This intervention is in line with the whole diet approach for MD, which has been shown to be more healthful compared to isolated nutrients [9]. As well as a whole of diet approach, the inclusion of cuisine means that the MD model intervention incorporates a combination of foods and optimises health outcomes based on the enhanced benefits of the food matrix and the preparation of these combinations.

Incorporating elements of cuisine such as combining EVOO with herbs and tomatoes resulted in the improved bioavailability of antioxidants and micronutrients; however, preparation may be considered challenging or not suitable for individuals who seek or require convenient options. To overcome this potential barrier the MD model intervention includes a range of non-cooking meal options for participants to ensure convenience, optimise consistency amongst dietary adherence and to enable sustainability. To date we are not aware of any direct comparison surrounding the effects of cuisine in cooked and raw meals. It is also likely that given the complexity around food and its constituents, results would be variable across specific foods and antioxidants.

One of the other key predicted barriers with application of a MD in non-Mediterranean countries, such as Australia, is the adaptability to other cultural preferences. This has been highlighted as a perceived barrier in other countries who have considered the application of a MD for the prevention and management of chronic diseases [75]. This paper showcased some practical solutions surrounding how to apply MD principles within other cultural dishes. In particular, the demonstration of the commonalities between ingredients and combinations from traditional dishes from range of countries was presented and how the composition and cuisine could be optimised with only minor recipe changes.

## 5. Conclusions

The MD model intervention maintained traditional dietary components to ensure that reported health benefits could be preserved while the context was adapted and evolved to suit an Australian multiethnic population. There were multiple factors that were considered, and thus, steps were developed to include strong evidence base and strategies surrounding potential barriers were applied to achieve this. Firstly, an optimal evidence-based dietary prescription which encompassed nutrition composition, ingredients and food combinations which were likely to elicit health benefits was adopted. Next, the dietary prescriptions were designed in a way that could be implemented across multiple cultures. This MD model is currently being assessed for acceptability and feasibility for participants with chronic diseases in two clinical trials. This MD model intervention demonstrates that it is possible to translate the key elements of the traditional MD to populations outside the Mediterranean region to increase the likelihood of acceptability and sustainability. Furthermore, this paper indicates the need for a consistent definition to describe the MD to ensure the key elements are captured and translated so that the translated MD model retains authenticity. The MD model and accompanying resources described herein can be used by nutrition researchers for future clinical trials adopting a MD. In addition, they may be used by health practitioners in the care of their patients with chronic diseases and by policy makers to provide evidence-based food guidelines for the prevention and management of many chronic diseases.

## Figures and Tables

**Table 1:** Nutrient data from the seminal Keys study and subsequent clinical trials within Mediterranean regions and clinical trials using a Mediterranean diet in Australian populations.

Trials	7 Countries Study	Diabetes Cross Over	PREDIMED	The Medi-RIVAGE	NAFLD Cross Over
	Keys et al. [15] (Cohort)	Itsiopoulos et al. [19] (Intervention)	Estruch et al. [20] (Intervention)	Vincent et al. [21] (Intervention)	Ryan et al. [4] (Intervention)
Dietary data	Prospective cohort	Feeding trial, full provision of diet. Data is recommended diet ^	Data is from diet consumed *	Data is from recommended diet ^	Feeding trial, full provision of diet. Data is the recommended diet ^
Population	Mediterranean	Non-Mediterranean (Australia)	Mediterranean (Spain)	Mediterranean (Spain)	Non-Mediterranean (Australia)
Nutrients					
Energy (MJ)	-	11.9	9.2	-	11.3
Protein (%E)	10.5	12.0	16.3	12–15	15.8
CHO (%E)		44.3	40.1	50	33.6
Total Fat (%E)	36.1	40.2	41.3	35–38	44.3
SFA (%E)	7.7	7.5	9.3	8–10	13.6
MUFA (%E)	25.8	22.9	21.5	18–20	22.8
PUFA (%E)	2.5	5.6	6.9	8–10	7.9
Alcohol (%E)	4	4	-	≤5%	1.5
Fibre (g/d)	-	46.7	-	>25 g	36.4
Linoleic acid *n*-6 (g)	-	15.6	14.1	-	15.1
α-linolenic acid *n*-3 (g)	-	1.5	1.6	-	1.6
EPA (g)	-	0.44	-	-	-
DHA (g)	-	0.48	-	-	-
Total LCN3s (mg)	-	-	-	-	200.3
Key outcome	All-cause mortality CHD	HbA1c	↓CVD complications	↓CVD risk	liver fat insulin resistance

Abbreviations: PREDIMED: Prevención con Dieta Mediterráne, Medi-RIVAGE: Mediterranean Diet, Cardiovascular Risks and Gene Polymorphisms, CHO: carbohydrates; SFA: saturated fatty acids; MUFA: monounsaturated fatty acids; PUFA: polyunsaturated fatty acids; EPA: eicosapentaenoic acid; DHA: docosahexaenoic acid; LCN3s: long chain omega 3 fatty acids; CHD: coronary heart disease; CVD: cardiovascular disease; NAFLD: non-alcoholic fatty liver disease. -: indicates that values were not published and/or measured; * The PREDIMED trial included two Mediterranean Diet (MD) arms; one with the provision of extra virgin olive oil (EVOO) and the other with nuts. Results presented are a mean of the consumption data presented from the two groups; ^ Recommended diet refers to the diet prescribed; therefore, a participant would receive the described macro- and micronutrient composition if they were 100% adherent.

**Table 2:** Nutrient composition of the Australian Mediterranean Diet *.

Nutrients	Australian Mediterranean Diet Composition
Energy (MJ)	9.4
Protein (%E)	15.8
CHO (%E)	33.8
Added sugar (%E)	5.2
Total fat (%E)	41.8
SFA (%E)	8.9
MUFA (%E)	22.3
PUFA (%E)	10.6
Alcohol (%E)	2.4
Fibre (g/d)	41.1
Linoleic acid *n*-6 (g)	18.7
α linolenic acid *n*-3 (g)	4.9
Total LCN3s (mg)	932

* This nutrient profile was calculated by entering two-week food diaries into the software program Foodworks 7™ (Xyris software Australia Pty Ltd.). Abbreviations: CHO: carbohydrates; SFA: saturated fatty acids; MUFA: monounsaturated fatty acids; PUFA: polyunsaturated fatty acids, LCN3s: long chain omega 3 fatty acids.

**Table 3:** The 12 components of a Mediterranean diet and the proposed mechanisms of effect.

Recommendation	Practical Dietary Applications	Key Components	Evidence Based Benefits
Use extra virgin olive oil (EVOO) as the main added fat.	Minimum 3–4 tablespoons (60–80 mL) per day	The highest proportions of MUFAs and polyphenols squalene and α-tocopherol are available in extra virgin olive oil.	Prevention of CHD, cancers and modification to immune and inflammatory responses have been attributed to EVOO. The high antioxidant content of EVOOs contributes to health of the vascular system through improved endothelial function [34] and has been shown to inhibit LDL oxidation [35]. EVOO consumption has also been proposed to improve bone mineralisation [36].
Eat vegetables with every meal.	Include 100 g leafy greens, and 200 g all other vegetables daily (cauliflower, zucchini, eggplant, capsicum etc.). use onion and garlic daily; include 100 g tomatoes daily; fresh or sofrito (tomato-based sauce).	Vegetables are the most significant source of phenolic compounds. They contain carotenoids, folic acid, fibre and phytosterols. Garlic, onion, herbs and spices also have key benefits. See Section 3.3.2 for importance of combining and/or cooking ingredients.	Flavonoids, are essential bioactive compounds that provide health benefits due to their antioxidant effects and have been associated with improvements in cognitive function and mood [37]. Traditional diets which are predominantly plant-based are associated with lower rates of chronic diseases and increased longevity [38]. Carotenoids, folic acid and fibre play important roles in CHD prevention [38]. Phytosterols contribute to reduced serum cholesterol and CVD risk [39]. Garlic, onions, herbs and spices contain large amounts of flavonoids or allicin which have cardiovascular benefits and also improve cognitive function [40]. Vegetables which are high in potassium, magnesium and calcium tend to reduce arterial blood pressure [34,36].
Include at least two legume meals per week.	Canned or dry legumes are acceptable; this may include tofu (1 serve = 250 g). This should replace meat on days when meat is not consumed.	Legumes are high in fibre, protein, B vitamins, iron, zinc, calcium, magnesium, selenium, phosphorus, copper and potassium [41]. They provide a nutritious, nourishing meat alternative.	Legumes are linked to longevity, and are a strong predictor of survival [42]. Vegetables have been shown to reduce serum homocysteine concentrations and thus coronary events, especially in high risk individuals [43]. An inverse association between the risk of T2DM and CHD and legume intake has been reported [44,45].
Eat at least three servings of fish or shellfish per week.	Fish (1 serve = 100–150 g); shellfish (1 serve = 200 g). Include oily fish at least 1–2 times per week.	Marine long chain omega-3 polyunsaturated fatty acids provide eicosapentaenoic acid (EPA) and docosahexaenoic acid (DHA).	EPA and DHA effectively regulate haemostatic factors and protect against cardiac arrhythmias, cancer and hypertension and help to maintain neural functions [34,40]. A high intake of fish and seafood has also been shown to reduce systolic blood pressure [34,36]. Immunomodulatory effects may improve inflammatory conditions [46].
Eat red meat less often and choose smaller portions. Choose white meat.	150–200 g weekly of beef, lamb and, pork. 200–250 g per week of poultry. Choose lean varieties, wild, free range and grass fed varieties are encouraged.	Meat is a bioavailable source of vitamin B12, and iron, selenium, and zinc and are a good source of protein. Excessive amounts of red meat have been linked to adverse health outcomes and excess saturated fatty acids—unfavourable fat ratios and displacement of more nutritious alternatives. Wild, free range and grass fed varieties are preferred due to the improved *n*-6:*n*-3 ratio [29].	Red meat is a good source of protein which assists with satiety [47]. Red and processed meats, especially when consumed in excess, are associated with total CVD and cancer mortality [48]. Excessive SFA intake from meat is also linked to adverse health outcomes [1].
Eat fresh fruit every day.	300g or 2 serves.	Fruit provides fibre, potassium, vitamins A and C, B vitamins, folate, flavonoids and terpenes providing protection against oxidative processes.	Consumption of fruit has been shown to reduce the risk of CVD and cancers [49]. Fibre, vitamins, minerals, flavonoids and terpenes may provide protection against oxidative processes which drive the onset and exacerbate chronic diseases [40]. Flavonoids have also been associated with improvements in cognitive function and mood [37].
Eat a serve of nuts every day and dried fruit as a snack or dessert.	Nuts-1 serve = ~30 g or 1/4 cup or a small handful daily. Dried fruit—2 tablespoons or 30 g.	Nuts are a good source of monounsaturated fats, fibre, vitamin C and E, selenium, magnesium, providing an abundance of antioxidants including flavonoids, resveratrol, polyphenols and tocopherols [50].	Monounsaturated fats, phenols, phytosterols, phytic acid and fibre are abundant in nuts and are associated with a reduction in plasma lipids and reduced incidence of CVD [40]. Nut consumption has been associated with the prevention and reversal of oxidative stress [50,51].
Eat dairy every day.	2 serves per day including milk-1 serve = 250 mL or 1 cup. Yoghurt preferably Greek style yoghurt 1 serve = 150 g or ¾ cup.	Dairy is a good source of calcium, vitamin D, phosphorus, magnesium, zinc, potassium, vitamins A and B12, and lactic acid bacteria confer probiotic effects [52]. Choose mostly fermented dairy which are higher in potent beneficial bioactive compounds from milk such as lactic acid bacteria [53].	Bioactive milk components have been shown to be protective in several diseases, including hypertension, coronary vascular diseases, obesity, osteoporosis and cancer [53]. Lactic acid bacteria confer probiotic effects, including improvements in gastrointestinal health and immune response. Yogurt, specifically, may induce desirable changes in faecal bacterial flora, potentially reducing the risk of colon cancer. Yoghurt is also likely to regulate mouth-to-caecum transit time [54].
Eat cheese in moderation, about 3 times per week and preferably feta.	1 serve = 30 g or the size of a matchbox.		
Include wholegrain breads and cereals with meals, such as wholegrain bread, rice, pasta and potato.	1 serve = 1 slice of bread or; ½ cup or; 50–60 g cooked pasta/rice or; 1 small 100 g potato.	Wholegrains are a good source of fermentable carbohydrates including fibre, resistant starch, and oligosaccharides. They contain phytochemicals, antioxidants including trace minerals, phenolic compounds, lignans and B group vitamins including folate, vitamin E, minerals iron, magnesium, copper and selenium [55].	Components such as fibre, antioxidants and vitamins and minerals promote health and may be protective against cancer, CVD, T2DM and obesity [56,57]. Production of short chain fatty acids through indigestible carbohydrates promote reduced serum cholesterol levels and decrease cancer risk [58].
Have sweets or sweet drinks in moderate amounts and on special occasions only.	Preferably home made.	Homemade varieties have key ingredients that are encouraged in the MD, such as nuts, EVOO and milk and are less refined and lower in SFA.	Liver fat accumulation may be attributed, at least in part, to excess dietary sugar consumption, especially from fructose, which increases the levels of enzymes involved in hepatic de-novo lipogenesis [59,60].
Consume up to 3 eggs per week.	Free range or omega-3 varieties.	Eggs are a good source of protein, choline, selenium, vitamin B12, riboflavin, phosphorus and fat soluble vitamins A, D and E. They are a bioavailable source of carotenoids; lutein and zeaxanthin, [61]. Free range and omega-3 enriched varieties have higher amounts of omega-3 fatty acids [62].	The benefits of eggs, including the provision of protein and micronutrients including vitamins, minerals and carotenoids may prevent age related macular degeneration and some cancers [61]. A limit to egg consumption is set to achieve the desired fat ratios in line with other MD guidelines [23].
OPTIONAL Consume wine in moderation.	Choose red wine. Have 0–2 glasses per day, (100 mL per glass) and always with meals. Do not get drunk.	Red wine contains phenolic compounds with high antioxidant properties. For example, red wine has higher amounts of the stilbene polyphenol, resveratrol, compared with white wine [63].	Red wine provides polyphenols whose antioxidant activity may contribute to the cytoprotective effects. Resveratrol has been found to protect the heart and kidneys from ischaemia-reperfusion injury and has a likely positive effect on endothelial function (vasodilation) with prolonged moderate consumption [64,65].

**Table 4:** Replicating the cuisine in a traditional Cretan Mediterranean dish across different cultures and cuisines.

Cuisine	Greek (Cretan)	Middle Eastern	Indian	Chinese	Western
Meal	Fasolatha	Mujadara	Dhal	Mapo Tofu	Homemade Baked beans
Key ingredients	Legumes, onions, garlic, tomato, herbs, EVOO	Lentils, rice, onions, spices, EVOO	Lentils onion, garlic, tomatoes, ghee	Tofu, garlic scallions, peppers, ginger, soy sauce +/− pork, peanut +/− sesame oils	Legumes, onion, garlic, tomato, vegetable oil
Fat Modifications	-	-	Replace *part or all* added fat with EVOO		

Abbreviations: EVOO: extra virgin olive oil, -: indicates no change was made or required.

## Supplementary Materials

The following are available online at http://www.mdpi.com/2072-6643/10/4/465/s1, Figure S1: Mediterranean Diet: two week cycle menu, Figure S2: Mediterranean Dietary Guidelines- Summary, Figure S3: Mediterranean Diet Pyramid, Figure S4: “No Cooking” Meal Options, Figure S5: Shopping List.
