# Supplementary material for: A Mediterranean Diet Model in Australia: Strategies for Translating the Traditional Mediterranean Diet into a Multicultural Setting
Source: Nutrients. 2018 Apr 9;10(4):465. doi: 10.3390/nu10040465 (PMC5946250; doi:10.3390/nu10040465)
Supplement: Supplementary file 1 [file nutrients-10-00465-s001.pdf]

Figure S1: Mediterranean Diet: Two week meal plan

WEEK 1

	Monday	Tuesday	Wednesday	Thursday	Friday	Saturday	Sunday
**Breakfast**	Porridge (cooked rolled oats with skim milk) with honey cinnamon and berries<br><br>Herbal Tea	2 slices of Soy and Linseed bread with chopped tomato, onion, herbs, olive oil and lemon juice<br><br>Coffee	Greek style low fat yogurt with chopped fruit, honey, cinnamon and nuts walnuts/ almonds<br><br>Coffee	2 pieces of Soy and Linseed bread with poached/boiled egg sliced, avocado a squeeze of lemon juice and cracked pepper<br><br>Herbal Tea	Porridge (cooked rolled oats with skim milk) cinnamon, bananas and honey<br><br>Herbal Tea	2 slices of Soy and Linseed bread with poached eggs in tomato stew<br><br>Coffee	Greek style low fat yogurt with chopped fruit, honey and cinnamon.<br><br>Coffee
**Snack**	Small handful of Walnuts	Low fat Greek yogurt with honey	Very small handful of Sultanas<br>Sesame snack bar	Low fat Greek yogurt with berries (fresh/frozen) and honey	2 slices of watermelon	Low fat Greek yogurt with honey	2 slices of honeydew
**Lunch**	Soy and linseed sandwich with spinach, tinned tuna in olive oil, tomato and onion	Left over Lentil soup drizzled with olive oil with 2 slices of soy and linseed bread and a piece of feta<br><br>Piece of fruit	2 slices of toasted soy and linseed bread with canned sardines in olive oil, with tomato and onion	Left over baked risoni with lamb and salad (lettuce cucumber and spring onion) dressed in olive oil.<br>Slice of Soy and linseed bread	Soy and linseed sandwich with feta cheese and roasted vegetables drizzled with olive oil	Left over Cannellini bean soup (white bean soup) with a slice of grain bread<br><br>Piece of fruit	Salad (pear, walnut and rocket) dressed with olive oil with tinned salmon and 2 slices of soy and linseed bread<br><br>Piece of fruit
**Snack**	Pear	Small handful of Almonds with 2 dried figs	Grapes	3 Dried apricots with a small handful of almonds	Small handful of Walnuts	Small handful of Almonds	Small handful of Walnuts
**Dinner**	Lentil soup drizzled with olive oil with 1 slice of soy and linseed bread and a piece of feta<br><br>Glass of wine	Baked snapper with baked potato and boiled greens with olive oil and lemon juice<br><br>Glass of wine	Baked risoni with lamb and salad (lettuce cucumber and spring onion) dressed in olive oil<br><br>Glass of wine	Chicken soup with egg and lemon<br>Carrot and coleslaw salad With a slice of soy and linseed bread<br><br>Glass of wine	Cannellini bean soup (white bean soup) drizzled with olive oil with a slice of soy and linseed bread<br><br>Glass of wine	Stewed chicken livers* with caramelised red onion in a red wine and oregano sauce, boiled greens with olive oil and lemon juice and with 2 slices of soy linseed bread<br>**can replace chicken livers with chicken fillet*<br>Glass of wine	Green pea casserole with beef<br>Rice pilaf with Greek coleslaw salad<br><br>Glass of wine
**Snack**	3 Dry figs	Two slices Rockmelon	Very small handful of Walnuts and a mandarin	Risogalo (rice pudding)	Orange	3 Dried figs	Piece of baklava

WEEK 2

	Monday	Tuesday	Wednesday	Thursday	Friday	Saturday	Sunday
**Breakfast**	Porridge (cooked rolled oats with skim milk) honey, cinnamon and berries<br><br>Herbal Tea	2 slices of Soy and Linseed bread with chopped tomato, onion, herbs, olive oil and lemon juice<br><br>Coffee	Greek style low fat yogurt with chopped fruit, honey, cinnamon and nuts (walnuts/ almonds)<br><br>Coffee	2 slices of Soy and Linseed bread with ricotta cinnamon and honey<br><br>Piece fruit<br><br>Herbal Tea	Porridge (cooked rolled oats with skim milk) honey, cinnamon and bananas<br><br>Herbal Tea	2 slices of Soy and Linseed bread with poached eggs, spinach and tomato dressed with olive oil and cracked pepper<br><br>Coffee	Greek style low fat yogurt with chopped fruit, honey and cinnamon<br><br>Coffee
**Snack**	Small handful of Walnuts	Low fat Greek yogurt with honey	<u>Very</u> small handful of sultanas<br>Sesame snack bar	Low fat Greek yogurt with berries (fresh or frozen)	2 slices of watermelon	Low fat Greek yogurt with honey sprinkled with walnuts	Slice of honeydew
**Lunch**	Soy and linseed sandwich with spinach, tinned salmon in olive oil, tomato and onion	Salad (spinach and rocket, tomato, cucumber, capsicum) with tinned 4 bean mix, herbs and feta cheese dressed with olive oil and vinegar<br>2 slices soy linseed bread<br>Piece of fruit	2 slices of toasted soy and linseed bread with canned sardines, olive oil, tomato and onion	Leftover Beef and cauliflower casserole with salad (lettuce cucumber and spring onion) dressed in olive oil and slice of Soy and Linseed bread	Soy and linseed sandwich with feta cheese and roasted vegetables sprinkled with olive oil	Salad (pear, walnut and rocket) dressed with olive oil with tinned tuna and 2 slices soy linseed bread<br><br>Piece of fruit	Left over Stuffed tomatoes with rice and bean salad, dressed in olive oil
**Snack**	Pear	Dried figs with a small handful of almonds	Grapes	Apple and small handful of walnuts	Small handful of Almonds	Apple	Small handful of Walnuts
**Dinner**	Baked (small) chicken breast*with broccoli and salad both dressed in olive oil<br>With couscous<br>*(recipe not supplied)	Baked salmon with boiled greens and beetroot salad dress in olive oil<br><br>Baked potato<br>Glass of wine	Beef and cauliflower casserole with salad (lettuce, tomato, cucumber and spring onion) dressed in olive oil and a baked potato<br><br>Glass of wine	Vegetable bake with Greek salad and feta<br><br>Glass of wine	Chicken casserole with onions and wine, rice and boiled warm wild greens dressed in olive oil and lemon juice<br><br>Glass of wine	Stuffed tomatoes with rice and black eyed bean salad, dressed in olive oil<br><br>Glass of wine	Eggplant moussaka with lamb mince and Greek coleslaw salad<br>1 slice Soy and Linseed bread<br><br>Glass of wine
**Snack**	3 Dry figs	2 slices of Rockmelon	<u>Very</u> small handful of Walnuts with a kiwi fruit	Risogalo (rice pudding)	Pear	3 dried figs and a very small handful of almonds	Piece of baklava

Guidelines to assist you in following the meal plan.

Main meals

- If you do not like one of the meals on the meal plan you may substitute it for another meal which cooks with the same protein, e.g. if you don't like salmon you may have another type of fish or seafood.

Sides

- If you wish to replace a side for something else you must substitute it for the same category of food (vegetables for vegetables, do not swap vegetables for a carbohydrate), e.g. if you don't like broccoli you may instead have another source of leafy greens or vegetables, however you must not replace it for rice.

Left Overs

- Many of the meals make 4-6 serves; these can be frozen to avoid having to cook every time. This especially works well for soups which make many serves and freeze very well. ***OR***
- You may have left over meals from dinner for lunch the following day, ensure that you include the salad/vegetable sides.

Fruit

- Where there is a serve of fruit listed you may choose a serve of any fruit that you enjoy/ have available, e.g. 1 apple, 1 orange, 1 banana, a handful of grapes, a slice of melon etc.

Yogurt

- Where Greek yogurt is listed as a snack you may have it plain or with nuts and honey or with some fresh or dried fruit. Add cinnamon for flavour.

Nuts

- All nuts should be raw and unsalted.
- Almonds and walnuts are preferred but all varieties are acceptable.

Portions

- Some of the meals have recommended portion sizes, these are just a guide. You should eat until you feel satisfied; this may be more or less of the suggested amount of food.

Copyright © 2017 by E.S. George & T. Kucianski
All rights reserved. This resource or any portion thereof may not be reproduced or used in any manner whatsoever without acknowledgment.

LA TROBE UNIVERSITY

Figure S2: Mediterranean Dietary Guidelines – Summary

1. **Use extra virgin olive oil as the main added fat** – *aim for minimum 3-4 tablespoons (60-80ml) per day*

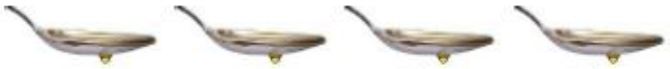

2. **Eat vegetables with every meal** – *include 100g leafy greens, and 200g other vegetables daily; use onion and garlic daily; include 100 g tomatoes daily; fresh or sofrito (tomato based sauce).*

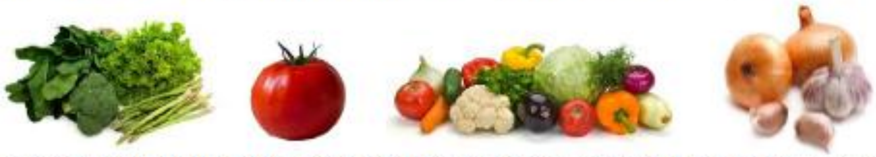

Leafy green veggies 1½ cups (raw) or ¾ cup cooked, 1 medium tomato or one tomato based meal, 2½ cups other veggies (raw) or 1½ cups other veggies (cooked), include onion/garlic in at least one meal per day (raw or cooked).

3. **Include at least two legumes meals per week** **canned or dry legumes are acceptable, this may include tofu (1 serve = 250 g.* This should replace meat.

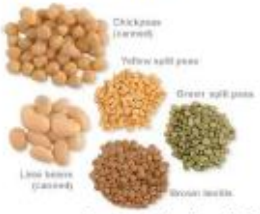

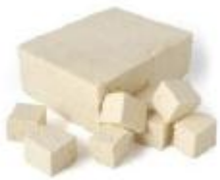

One serve is about half a standard can or 1½ cups (cooked).

4. **Eat at least three servings of fish** *(1 serve = 100-150 g)* **or shellfish** *(1 serve = 200g)* **per week** – *include oily fish at least 1 – 2 times per week.*

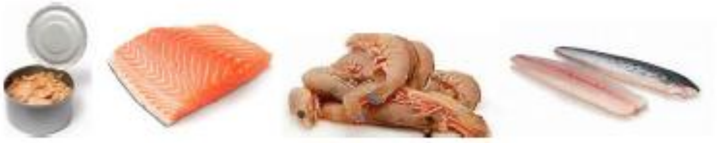

5. **Eat red meat less often and choose smaller portions** *(150-200g weekly) (beef, lamb, pork).*
Limit white meat *(200 – 250g per week) (poultry).* **Choose lean varieties.**

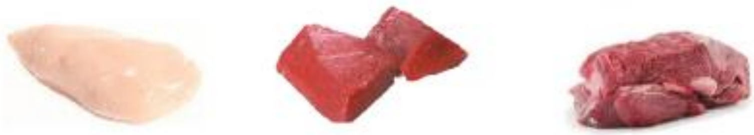

*The recommended serving of 'meat' can be eaten in one meal or over multiple, as long as you do not exceed the maximum allowance per week.

Copyright © 2017 by E.S. George & T. Kucianski
All rights reserved. This resource or any portion thereof may not be reproduced or used in any manner whatsoever without acknowledgment.

LA TROBE UNIVERSITY

6. Eat fresh fruit every day *(300g or 2 serves).*

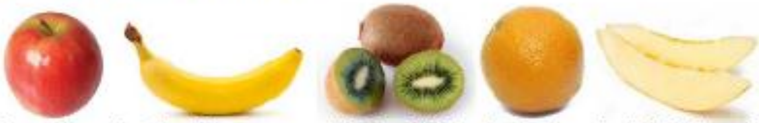

*<u>1 serve</u> is equivalent to: 1 large fruit (apple, banana, orange) OR 2 small fruits (kiwi, apricot, plum) OR 2 slices of fruit (watermelon, rockmelon, pineapple) OR 15 grapes or cherries.

7. Eat a serve of nuts every day (1 serve = ~30g or 1/3 cup or a small handful daily) and dried fruit *(2 tablespoons or 30g)* as snacks or dessert;

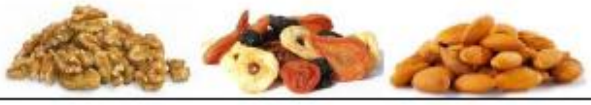

8. Eat dairy every day (2 serves) including milk *(1 serve = 250ml or 1 cup)* and yoghurt *preferably Greek style yoghurt (1 serve = 150g or ¾ cup)*;

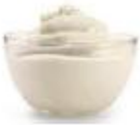

9. Eat cheese in moderation, *about 3 times per week and preferably feta (1 serve = 30g or the size of a matchbox)*;

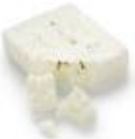

10. Include wholegrain breads and cereals with meals *such as wholegrain bread, rice, pasta and potato (1 serve = 1 slice of bread or ½ cup, 50-60g cooked pasta/rice or 1 small 100g potato)*

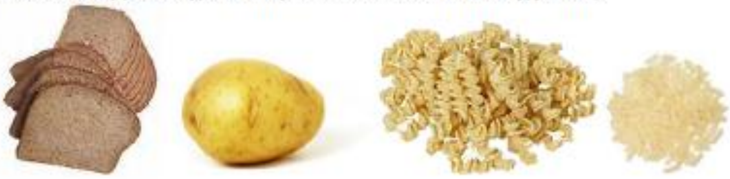

11. **OPTIONAL** Consume wine in moderation *(preferably red), (0-2 glasses, 100ml per glass) and always with meals and don't get drunk.*

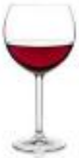

12. Have sweets or sweet drinks in moderate amounts *and on special occasions only.*

13. Consume no more than 3 eggs per week *and select free range or omega 3 varieties.*

Copyright © 2017 by E.S. George & T. Kucianski
All rights reserved. This resource or any portion thereof may not be reproduced or used in any manner whatsoever without acknowledgment.

LA TROBE UNIVERSITY

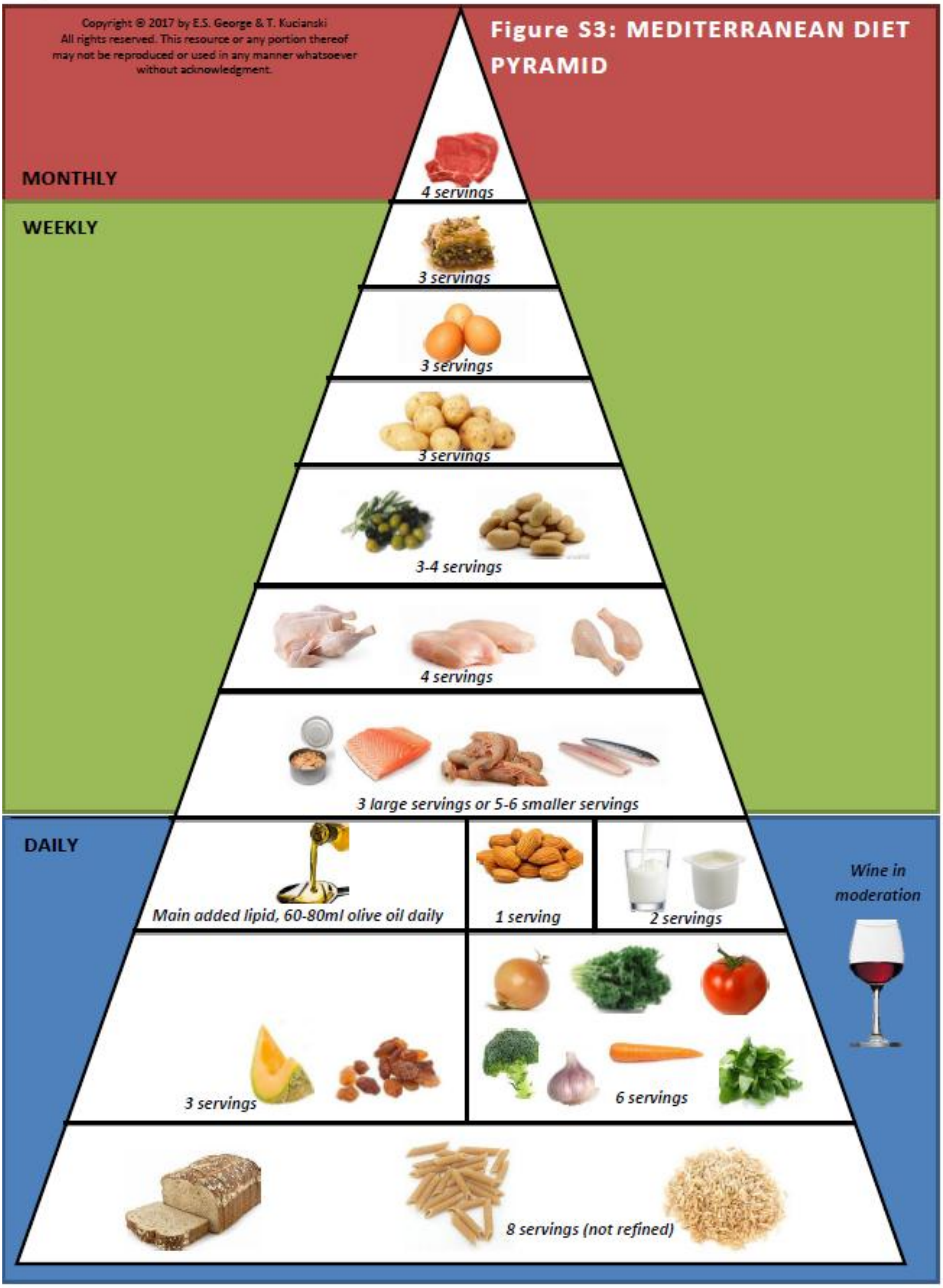

Figure S3: MEDITERRANEAN DIET PYRAMID

Figure S4: No Cooking Meal options

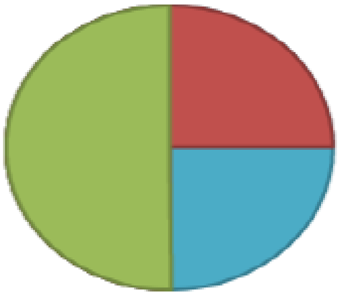

"No Cooking" Meal Options

Protein

Canned Beans; 4 bean mix, chickpeas, kidney beans, cannellini beans, brown lentils,

Canned Fish; tuna in oil or water, salmon, sardines, trout, mackerel

Canned seafood; oysters, octopus, mussels,

Canned Chicken; in oil or water

Nuts; almonds, walnuts, hazelnuts, whole or spreads

+

Veggies/salad

Salad: Salad mix, Lettuce tomato and cucumber, cabbage and carrot shredded, spinach and tomatoes, beetroot and rocket, celery carrot and capsicum.

Vegetables: Frozen veggies (mixed or individual varieties), left over veggies from dinner.

There are just examples and there are many other possible variations.

+

Carbohydrate

Whole grain crackers

Soy and Linseed bread; plain or toasted

Whole grain wrap; plain or toasted

Rice; Brown rice or brown rice and quinoa sachet can be microwaved, left over from dinner

Couscous; pour enough boiling water to coat grains in a bowl or container, these will be ready to eat in 2 minutes.

Potato (white or sweet); left over from dinner or cooked in the microwave

Pasta; left over from dinner

Noodles; fresh varieties cooked by coating with boiling water for 5 minutes and draining

LA TROBE UNIVERSITY

Copyright © 2017 by E.S. George
All rights reserved. This resource or any portion thereof may not be reproduced or used in any manner whatsoever without acknowledgment.

Figure S5: Shopping List

Shopping List

Required Ingredients for the two week menu supplied.

Vegetables

Onions (brown and red)
Garlic
Spinach
Lettuce (cos, rocket)
Cucumber
Spring onions
Rocket
Potatoes
Wild Greens- endive, chicory, dandelion, sow thistle or mixed leafy greens
Eggplant
Frozen peas
Carrots
Cabbage
Capsicum
Green beans
Broccoli
Beetroot (canned ok)
Celery
Zucchini
Cauliflower

Grains

Rolled Oats
Soy and Linseed Bread
Rice
Risoni pasta
Lentils green or brown (dry)
Cannellini beans (dry or canned)
Black eyed beans (dry)
Canned 4 bean mix

Dairy

Skim milk
Greek style Low Fat yogurt
Feta cheese
Reduced fat ricotta

Beverages

Herbal Tea
Red Wine
White wine (for cooking)

Fruit

Frozen berries
Tomatoes
Lemon
Avocado
Pears
Apples
Grapes
Bananas
Melon (honey dew/ rockmelon/ watermelon)
Oranges
Mandarin

Poultry/ Seafood / Meat

Eggs
Tuna tinned in olive oil/spring water
Salmon tinned in olive oil/spring water
Sardines canned
Snapper, whole
Skinned chicken pieces
Lamb loins (lean)
Chicken breast
Salmon
Lamb Mince (lean)
Lean Beef

Other

Olive oil
Spices such as cinnamon, nutmeg, bay leaf, chilli flakes, cloves
Mixed Herbs such as oregano, parsley, thyme
Vinegar- balsamic
Walnuts
Almonds
Sultanas
Dry figs
Honey
Pureed tomatoes
Currents
Sesame snack bars
Breadcrumbs

LA TROBE UNIVERSITY

Copyright © 2017 by E.S. George & T. Kucianski
All rights reserved. This resource or any portion thereof may not be reproduced or used in any manner whatsoever without acknowledgment.
